# Genetic differentiation and admixture between sibling allopolyploids in the Dactylorhiza majalis complex

**DOI:** 10.1038/hdy.2016.119

**Published:** 2016-12-07

**Authors:** F Balao, M Tannhäuser, M T Lorenzo, M Hedrén, O Paun

**Correction to:** Heredity (2016) **116,** 351–361; doi:10.1038/hdy.2015.98; published online 25 November 2015

**Updated online 7 December 2016**: This article was originally published under a CC BY-NC-SA 4.0 license, but has now been made available under a CC BY 4.0 license. The PDF and HTML versions of the paper have been modified accordingly.

